# Sublethal Photothermal Stimulation with a Micropulse Laser Induces Heat Shock Protein Expression in ARPE-19 Cells

**DOI:** 10.1155/2015/729792

**Published:** 2015-11-30

**Authors:** Keiji Inagaki, Takuya Shuo, Kanae Katakura, Nobuyuki Ebihara, Akira Murakami, Kishiko Ohkoshi

**Affiliations:** ^1^Department of Ophthalmology, St. Luke's International Hospital, 9-1 Akashi-cho, Chuo-ku, Tokyo 104-8560, Japan; ^2^Department of Ophthalmology, Juntendo University Graduate School of Medicine, Hongo 2-1-1, Bunkyo-ku, Tokyo 113-8421, Japan; ^3^Institute for Medical Innovation, St. Luke's International University, 10-1 Akashi-cho, Chuo-ku, Tokyo 104-8560, Japan; ^4^Department of Ophthalmology, Juntendo University Urayasu Hospital, 1-1 Tomioka 2-chome, Urayasu-shi, Chiba 279-0021, Japan

## Abstract

*Purpose/Aim of the Study.* Subthreshold micropulse diode laser photocoagulation is an effective treatment for macular edema. The molecular mechanisms underlying treatment success are poorly understood. Therefore, we investigated the effects of sublethal laser energy doses on a single layer of densely cultured ARPE-19 cells as a model of the human retinal pigment epithelium (RPE).* Materials and Methods.* A single layer of densely cultured human ARPE-19 cells was perpendicularly irradiated with a micropulse diode laser. Nonirradiated cells served as controls. Sublethal laser energy was applied to form a photocoagulation-like area in the cultured cell layers. Hsp70 expression was evaluated using quantitative polymerase chain reaction and immunocytochemistry.* Results.* Photocoagulation-like areas were successfully created in cultured ARPE-19 cell layers using sublethal laser energy with our laser irradiation system.* Hsp70* mRNA expression in cell layers was induced within 30 min of laser irradiation, peaking at 3 h after irradiation. This increase was dependent on the number of laser pulses. Hsp70 upregulation was not observed in untreated cell layers. Immunostaining indicated that Hsp70 expression occurred concentrically around laser irradiation sites and persisted for 24 h following irradiation.* Conclusion.* Sublethal photothermal stimulation with a micropulse laser may facilitate Hsp70 expression in the RPE without inducing cellular damage.

## 1. Introduction

Laser photocoagulation is widely used for treating various retinal diseases, including diabetic retinopathy [1], branch retinal vein occlusion [2], and central serous retinopathy [3]. Unfortunately, conventional laser treatments are destructive procedures used to create whole-layer damage to retinal tissue. Such invasive treatments are not suitable for macular diseases, including macular edema, because laser scars generally grow larger and create permanent scotomas. Laser scar enlargement, subretinal fibrosis, and subretinal neovascular membranes have been reported as serious complications of laser treatment [4–8].

Micropulse lasers were invented to reduce laser-induced burns that can occur with conventional laser treatment [9]. Lasers in the micropulse mode exert pulses of short laser applications, thereby diminishing the heat generated at the target site [10]. In fact, retinal coagulation scars are not visible upon fundoscopic examination, autofluorescent imaging, or optical coherence tomography [11, 12] when the eyes are treated with a subthreshold micropulse laser. Many studies have shown the clinical efficacy of this treatment [10, 13–17]; however, few studies have investigated the molecular mechanisms responsible for fluid absorption from retinal tissue [18]. Although thermal stimulation of the retinal pigment epithelium (RPE) is thought to be the key trigger for fluid absorption from an edematous retina, the molecular mechanisms of this process are not yet understood. Notably, Lavinsky et al. found slight morphological changes following laser irradiation with a low-energy output in Dutch-belted rabbits [19]. These changes were reversible (i.e., they did not cause permanent damage) when photocoagulation spots (visible burns) were barely visible on the retinal surface. These data suggested that biological cascades within the RPE may contribute to the therapeutic effects of sublethal photocoagulation during the treatment of macular edema.

Heat shock protein (Hsp) family members, which act as chaperone proteins, aid in refolding denatured proteins and are protective against apoptosis and inflammation [20–24]. In particular, elevated expression of Hsp is known to occur with increases in temperature. Upregulation of Hsp70 expression induced by laser irradiation is thought to play an important role in the improvement of macular edema [25]. Intriguingly, nondamaging laser irradiation induces Hsp70 expression within the RPE of hemizygous C57BL/6 mice [26], whereas low-energy laser irradiation induces RPE cell death in Dutch-belted rabbits [19]. Unfortunately, whether sublethal laser energy induces Hsp70 expression in human retinal tissue is still unknown.

In this study, as a first step towards understanding the molecular mechanisms through which micropulse laser application affects retinal tissue, we constructed a laser irradiation system to directly expose cultured human retinal pigment epithelial ARPE-19 cell layers to a sublethal dose of laser energy. The expression of Hsp70 at both the mRNA and protein levels was examined over time.

## 2. Materials and Methods

### 2.1. Cell Culture

The ARPE-19 immortal human RPE cell line [27] at passage 19 (ATCC CRL-2303, Lot number: 60279299) was purchased from American Type Culture Collection (Manassas, VA, USA), and all experiments were performed using the cells at passages 22 through 26. Cells were maintained in Dulbecco's modified Eagle's medium: Nutrient Mixture F-12 (DMEM/F-12, Gibco, Life Technologies, Carlsbad, CA, USA) supplemented with 10% fetal bovine serum (One Shot FBS; Gibco, Life Technologies) and 1% penicillin-streptomycin (Pen Strep; Gibco, Life Technologies) at 37°C in a 5% CO_2_ incubator. Cell culture media were replaced 2-3 times a week. For laser applications, 3 × 10^5^ cells were seeded on the glass bottoms (12 mm in diameter) of 35 cm^2^ glass-bottomed culture dishes (Asahi Techno Glass, Tokyo, Japan) and left to stand for 30 min. Two milliliters of DMEM/F-12 was then added, and media were replaced on the fourth day of culture. Fully confluent cells were used in experiments on the eighth day of culture.

### 2.2. Laser Application

Diode lasers are primarily used for targeting of RPE cells in photocoagulation treatment in the clinical setting. Therefore, we constructed an experimental system that made it possible to irradiate a fully confluent layer of cultured cells. In order to directly irradiate cultured cell layers with a laser, as is performed in the clinical setting, a slit-lamp adapter for an 810 nm diode laser (Oculight SL; Iris Medical, Mountain View, CA, USA) was placed on top of a slit-lamp system created by cutting a slit-lamp microscope barrel (900 BM; Haag-Streit Diagnostics, Bern, Switzerland) and vertically fixing it onto a culture dish. After laser irradiation, cells were inspected with a confocal microscope (TCS SP8; Leica Microsystems, Mannheim, Germany) equipped with a life cell imaging chamber (Stage Top Incubator; Tokai Hit, Shizuoka, Japan) and/or a phase-contrast microscope (DMI3000B; Leica Microsystems). Measurements of photocoagulated areas were performed using an image analyzer (LAS 4.2; Leica Microsystems) and Image J 1.47 public domain software (National Institutes of Health, Bethesda, MD, USA).

### 2.3. Cell Viability Assay

A LIVE/DEAD cell imaging kit (Molecular Probe, Life Technologies) was used to visualize live and dead cells after laser irradiation. The LIVE/DEAD reagent, composed of calcein-AM and ethidium homodimer-1, was directly added to conditioned media on cultured cells after laser irradiation and incubated for 15 min at 25°C. To discriminate between live and dead cells, the RPE cell layer was inspected with a confocal microscope with the appropriate filters in place. Live cells were able to hydrolyze calcein-AM and were therefore stained green, while dead cells were permeable to ethidium homodimer-1 and were therefore stained red.

### 2.4. Quantitative Reverse Transcription Polymerase Chain Reaction (qRT-PCR)

All RNA isolation experiments were performed using a PureLink RNA Mini Kit with DNase (Ambion, Life Technologies) according to the manufacturer's instructions. First-strand cDNA was synthesized from 200 ng of total RNA using a High Capacity RNA-to-cDNA Kit (Applied Biosystems, Life Technologies). For quantification of* Hsp70* mRNA, one-twentieth of the above-mentioned cDNA was analyzed using TaqMan Fast Advanced Master Mix (Applied Biosystems, Life Technologies) and TaqMan Gene Expression Assay probe-primer sets for* HSPA1A* (Hs00359163_s1; Applied Biosystems, Life Technologies). The level of* Hsp70* mRNA was determined by the 2^−ΔΔCT^ method [28] using* GAPDH* (Hs02758991_g1, Applied Biosystems, Life Technologies) as an internal control.

### 2.5. Immunocytochemistry

Cells were fixed with 4% paraformaldehyde (Thermo Fischer Scientific, Waltham, MA, USA) and permeabilized using 0.1% Triton X-100. Cells were treated with Image-iT FX signal enhancer (Molecular Probe, Life Technologies), blocked in 10% normal goat serum (Molecular Probe, Life Technologies), and incubated with an anti-Hsp70 antibody (3A3; Abcam, Cambridge, UK) or anti-GAPDH antibody (loading control; Abcam). These primary antibodies were visualized with Alexa Fluor 488 Goat Anti-Mouse IgG antibody (Molecular Probe, Life Technologies), which served as a secondary antibody. Samples were counterstained with SlowFade Gold Antifade Mountant with 4′,6-diamidino-2-phenylindole (DAPI; Molecular Probe, Life Technologies). Immunofluorescent images of cells were captured with a confocal microscope, as described above.

### 2.6. Statistical Analyses

Student's *t*-tests and Mann-Whitney *U* tests were used to test mean differences for statistical significance. Statistical calculations were performed using SPSS software (SPSS, Inc., Chicago, IL, USA). Each experiment was conducted more than three times. Differences with *p* values of less than 0.05 were considered statistically significant.

## 3. Results

### 3.1. Laser Irradiation System for Cultured Cell Layers

**Figure 1:**
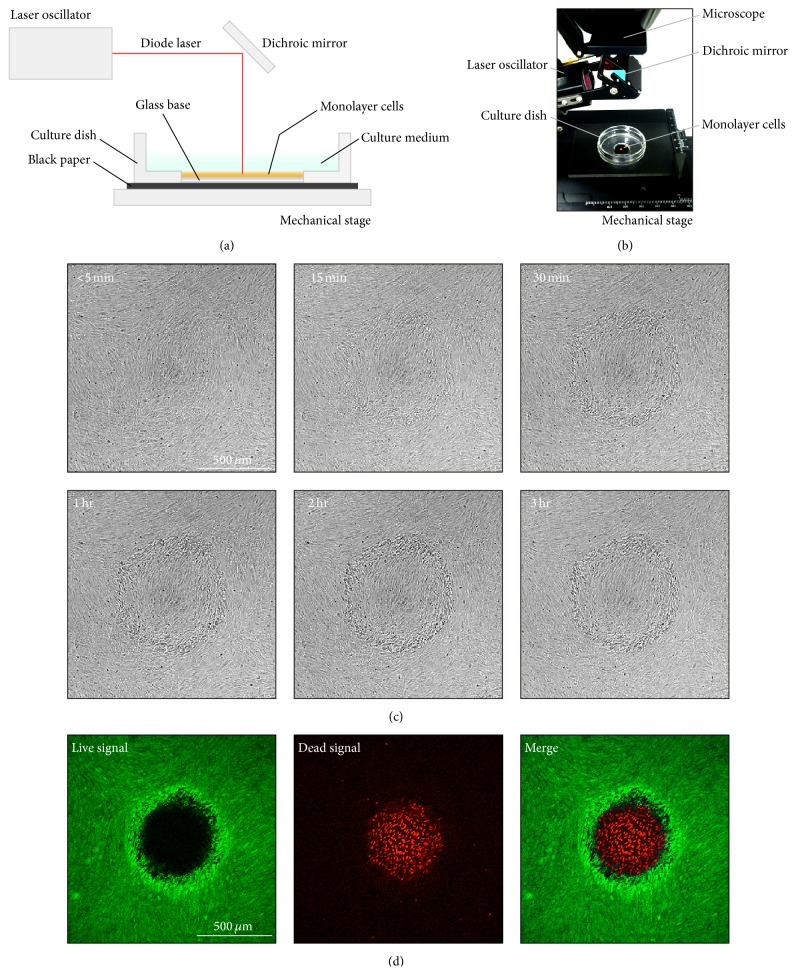
Experimental setup for laser irradiation of cultured cell layers. (a, b) A diode laser beam passed through a dichroic mirror to perpendicularly irradiate a full confluent cultured cell layer on a glass-based dish. To avoid blocking diode laser light, a phenol-red-free culture medium was used. (c) Confocal microscopy bright-field images of the irradiated cell layer. Spots were made using the continuous-wave mode with a power of 250 mW, duration of 500 ms, and spot size of 200 *μ*m. The white dotted circle indicates the laser irradiation site. A video is also available (see Supplementary Material 1). (d) Two hours after laser irradiation (using the laser settings described in (c)), cell viability was assessed using LIVE/DEAD cell imaging reagents. Live cells were stained green, and dead cells were stained red. Fluorescent images were captured with confocal microscopy (projected images). Cells within the laser spot were dead, but cell groups forming the sparse annular intercellular spaces observed in the bright-field images were alive.

Cells were irradiated on glass-bottomed dishes via a dichroic mirror and a perpendicular laser (Figures 1(a) and 1(b)). In order to uniformly expose the cell layer to laser energy, the culture dish was plated on black paper. Several methods of spreading black paper under culture dishes when performing laser irradiation in ARPE-19 cells have been previously reported [29, 30]. Additionally, culture media without phenol red were used to avoid blocking of the emitted diode laser light. The culture dish was placed on a mechanical stage so that the dish could be moved into the proper laser irradiation position in a flat *X*-*Y* plane. The stage was moved in 0.1 mm steps, and the cell layer was accurately irradiated with the laser in equal intervals. The position of the laser irradiation site was indicated by a red light pointing in the direction of the *X*- or *Y*-axis. The position along the *Z*-axis was then determined using the focal point of the laser. The focal point was defined as the position in which the laser aiming beam was most clearly projected onto the surface of the black paper.

When a cell layer was irradiated with a conventional continuous-wave mode laser, the laser irradiation site and its periphery gradually changed into a coagulation-like form. The formation of sparse annular intercellular spaces around the laser irradiation site was also observed in bright-field images obtained with a confocal microscope 2 h after laser irradiation (Figure 1(c) and Supplementary Material 1 in Supplementary Material available online at http://dx.doi.org/10.1155/2015/729792). These cell groups in the bright-field images remained viable, as indicated by green fluorescence staining with calcein-AM, an indicator of cell survival. In contrast, the laser irradiation site and nearby cells were dead, as indicated by red fluorescence staining with ethidium homodimer-1, an indicator of cell death (Figure 1(d)).

### 3.2. Morphological Changes in Cultured Cell Layers after Sublethal Micropulse Laser Irradiation

**Figure 2:**
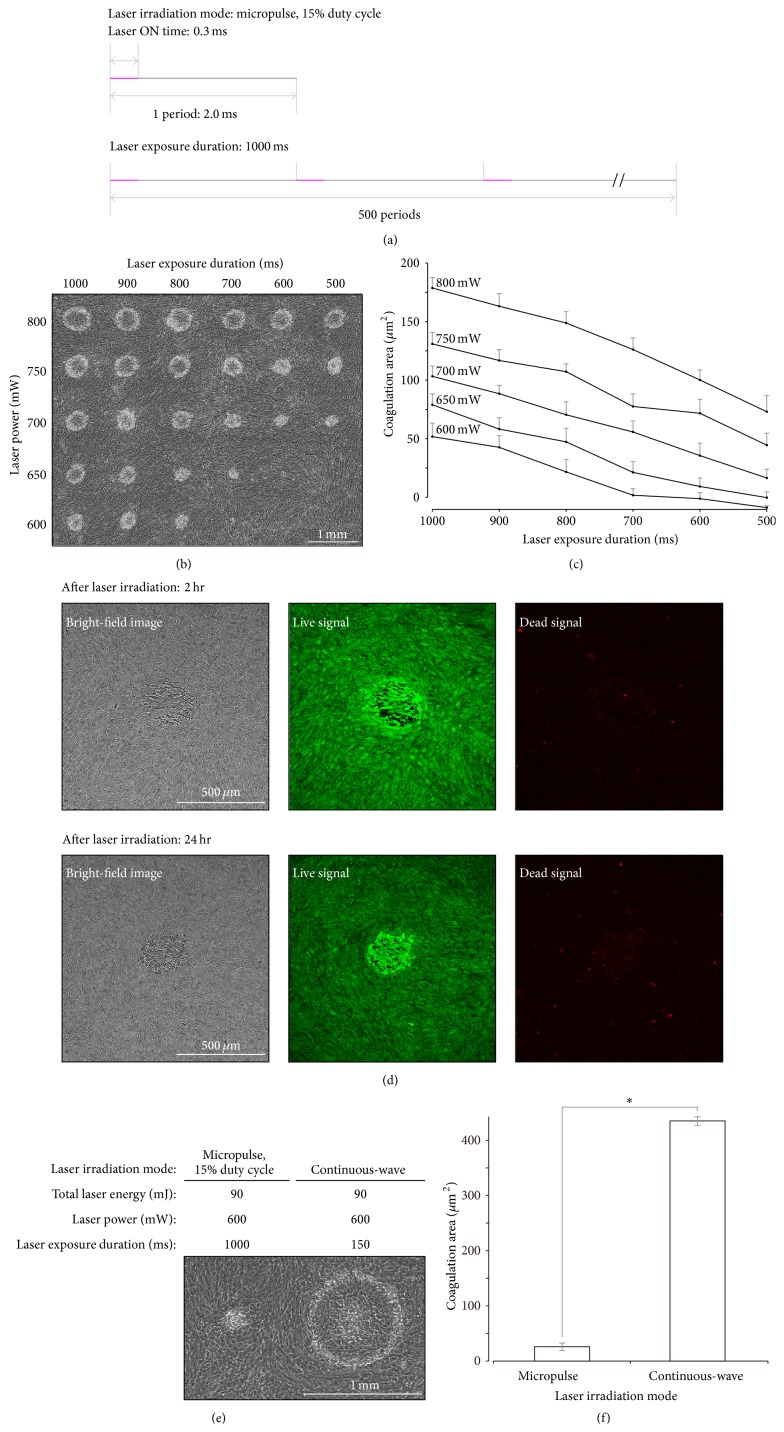
Establishment of laser parameters for sublethal photothermal stimulation on a cultured cell layer using micropulse-mode laser irradiation. (a) A micropulse-mode laser emits short, intermittent pulses of laser energy. At a 15% duty cycle, the laser was on for 0.3 ms during each 2.0 ms period. When the total laser exposure duration was 1000 ms, the laser cycle was repeated 500 times. (b) Representative phase-contrast microscopy images taken 2 h after laser irradiation with a micropulse laser. The laser had a 15% duty cycle and was set to various laser powers (600–800 mW) and laser exposure durations (500–1000 ms). Multiple spots were made on a single one-cell layer culture plate 1 mm apart in both the *X* and *Y* directions. The duration was varied along the *X*-axis, and power was varied along the *Y*-axis. The laser spot size remained constant at 200 *μ*m. (c) Areas of morphological change are shown in (b), as measured with NIH Image J software. Data represent the mean ± standard error of 10 independent experiments. Both laser exposure duration and laser power were significantly correlated with coagulation area (Spearman's correlation coefficient: *r* > 0.8, *p* < 0.05). (d) Representative bright-field (left panels) and fluorescent (center and right panels) images around laser irradiation sites, as captured with confocal microscopy, at the indicated times following laser irradiation. Sublethal photothermal stimulation was performed with the following laser settings: 15% duty cycle, 1000 ms laser exposure duration, and 600 mW laser power. Note that no dead cell signals (red fluorescence) were observed within the 24 h following laser irradiation. (e) Representative phase-contrast microscopy images 2 h after laser irradiation of single-cell layers with a laser in the micropulse (left) and continuous-wave (right) modes. The total laser irradiation energy was 90 mJ in both cases. (f) Mean coagulation area of spots made with a laser in the micropulse and continuous-wave modes shown in (e). Error bars represent one standard error (*n* = 12). The asterisk (*∗*) indicates a statistically significant difference between means (Mann-Whitney *U* tests, *p* < 0.01).

A laser provides intermittent irradiation in the micropulse mode, with the percentage of irradiation time set at a fixed number. This number is known as the duty cycle. In the micropulse mode, the duty cycle is 15%, with the laser irradiating for 0.3 ms during each 2.0 ms period. When the total micropulse laser exposure duration was 1000 ms, the duty cycle was repeated 500 times (Figure 2(a)).

Figure 2(b) shows phase-contrast images of irradiation sites 2 h after micropulse laser irradiation was performed with the following laser settings: duty cycle of 15%, laser exposure duration between 500 and 1000 ms, and laser power between 600 and 800 mW. Laser irradiation was performed at 1 mm intervals along the *X*- and *Y*-axes on each cell layer. Laser exposure duration was shortened with each 1 mm position change along the *X*-axis, but laser power remained constant. Laser power was decreased with each 1 mm position change along the *Y*-axis, but laser exposure duration remained constant. When the laser power was fixed at 800 mW, the coagulation area gradually decreased as the laser exposure time was shortened (1000 to 500 ms: 184 ± 8.8 to 81 ± 13.5 *μ*m^2^). However, coagulation-like lesions were no longer visible with phase-contrast microscopy when the laser power was 600 mW and the laser exposure duration was 600 ms. Coagulation area was significantly correlated with laser exposure duration. Additionally, coagulation area proportionally increased with laser power within the range examined (Spearman's correlation coefficient: *r* > 0.8, *p* < 0.05, Figure 2(c)).

Based on the observed changes in micropulse laser irradiation lesions in a cultured cell layer (Figure 2(b)), the optimal, reproducible sublethal laser settings were determined to be a laser exposure duration of 1000 ms, a duty cycle of 15%, and a laser power of 600 mW. Bright-field images, captured with a confocal microscope, revealed that cell morphology changed in sublethal photothermal stimulation sites where intercellular spaces were sparse (Figure 2(d)). However, almost all cells within sublethal photothermal stimulation sites were alive, even 24 h after micropulse laser irradiation (Figure 2(d)). Additionally, the coagulation-like area was significantly smaller for lesions created in the micropulse mode than for those created in the continuous-wave mode under the same energy conditions (Mann-Whitney *U* test: *p* < 0.001, Figures 2(e) and 2(f)).

### 3.3. Hsp Expression in Cultured Cell Layers following Sublethal Laser Irradiation

**Figure 3:**
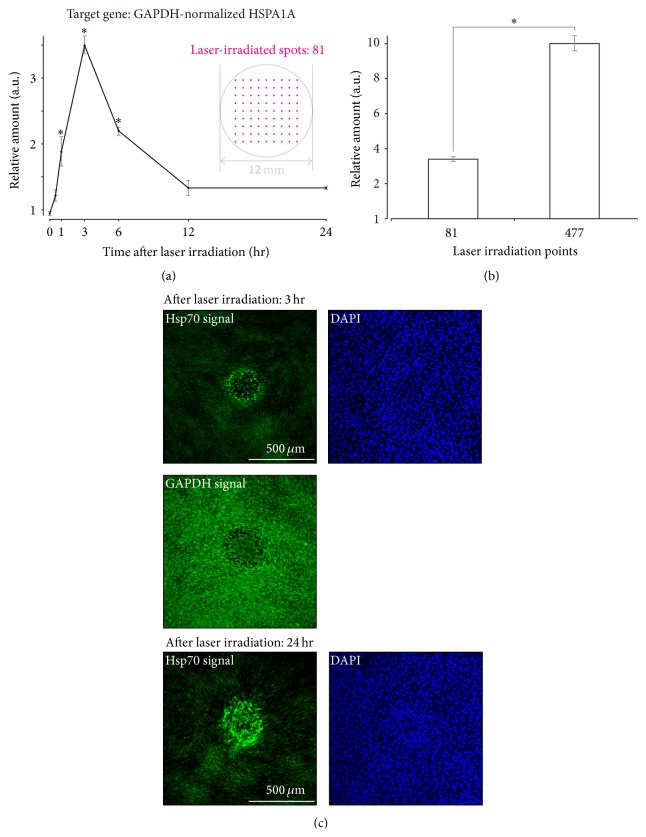
Changes in the expression of heat shock protein 70 (Hsp70) in cultured cell layers after sublethal photothermal stimulation. (a–c) Micropulse-mode laser irradiation sessions of cells on the glass-based dish were made using the following laser settings: 1000 ms exposure duration and 600 mW laser power. (a) Sublethal laser irradiation led to a transient upregulation of* HSPA1A* mRNA, which encodes the Hsp70 protein. At the indicated time points, total RNA was extracted, and* HSPA1A* mRNA levels were assessed using real-time quantitative reverse transcription polymerase chain reaction (qRT-PCR).* GAPDH* mRNA was used as an internal control. The data presented in the figure were normalized to the amount of* HSPA1A* mRNA in nonirradiated samples. Data represent mean values of three independent experiments. Error bars represent one standard deviation. The asterisk (*∗*) indicates statistical significance (*p* < 0.05), as determined using Student's *t*-tests. A schematic of the 81 laser irradiation spots on the cultured cell layer is shown in the insert. (b) Three hours following laser irradiation,* HSPA1A* mRNA upregulation was higher when 477 laser irradiation spots were made than when 81 spots were made. The asterisk (*∗*) indicates a statistically significant difference between means (*n* = 3, Student's *t*-test, *p* < 0.001). (c) Upregulation of Hsp70 protein at laser irradiation sites 3 h (upper and middle panels) and 24 h (lower panel) after sublethal photothermal stimulation. Cell layers were immunostained with the indicated antibodies and counterstained with 4′,6-diamidino-2-phenylindole (DAPI). Fluorescent images were captured with a confocal microscope (projected images). Immunofluorescent signals representing Hsp70 expression were detected around laser irradiation sites 24 h after laser irradiation (lower left).

To investigate whether sublethal photocoagulation induced Hsp expression, which is important in reducing macular edema, we used qRT-PCR to examine the expression levels of* HSPA1A* mRNA, which encodes Hsp70 protein, in a cultured cell layer irradiated with a sublethal irradiation dose using a micropulse laser (Figure 2(d)). Cells were examined until 24 h after irradiation from each of the 81 irradiation positions in the *X*-*Y* plane. In the pretreated irradiation group, maximum* HSPA1A* mRNA expression was observed 3 h after photothermal stimulation. The expression of* Hsp70* mRNA was only slightly increased over baseline, even 24 h after irradiation (Figure 3(a)). Because the clinical efficacy of macular edema treatment has been shown to be better with high-density micropulse laser irradiation than with standard laser treatment [16], we also examined the effects of varying the number of laser irradiation lesions on* HSPA1A* mRNA levels by changing the spacing of laser irradiation lesions on the cultured cell layer, which resulted in an increase in the number of lesions from 81 to 477. As expected, a significant increase in* HSPA1A* mRNA expression was observed 3 h after irradiation in cell layers with a higher number of lesions (Figure 3(b)).

Actual Hsp70 protein levels in cultured cell layers were also examined after sublethal laser irradiation using immunocytochemistry with an anti-Hsp70 antibody (Figure 3(c)). Hsp70 immunofluorescent signals were present in concentric areas around laser irradiation sites. However, GAPDH, used as an endogenous control for qRT-PCR analyses of* HSPA1A* mRNA, was uniformly present throughout the cultured cell layer. The presence of cells within laser irradiation sites was confirmed with DAPI counterstaining. Even 24 h after photothermal stimulation, Hsp70 immunostaining persisted at laser irradiation sites.

## 4. Discussion

In this study, we established a system in which one layer of densely cultured cells could be irradiated with a diode laser. We successfully and repeatedly created coagulation-like lesions without inducing cell death. Moreover, we confirmed that sublethal micropulse photothermal stimulation induced Hsp70 expression in a cultured layer of ARPE-19 cells.

Previous reports in the literature have shown that the size of coagulation lesions created using a continuous-wave laser is dependent on laser duration and power in Dutch-belted rabbits [31, 32]. Our in vitro study results are consistent with this and revealed that this is also true for coagulation-like lesions made with a micropulse laser (Figures 2(b) and 2(c) and Supplementary Material 2). Using the same amount of energy, more tissue damage occurred in lesions created with a continuous-wave laser than in those created with a micropulse laser (Figures 2(e) and 2(f)). It is thought that micropulse lasers cause less damage than continuous-wave lasers because the pulses of very short, intermittent energy may allow for heat dissipation within tissue. This would reduce tissue temperature elevation during laser application. Although numerous clinical studies have shown the efficacy of subthreshold micropulse laser treatments, the laser settings (i.e., laser power and pulse duration) used in these studies have not been consistent [10, 13–17]. Moreover, the optimal laser energy for subthreshold laser treatments has not yet been established, and surgeons often use continuous-wave laser modes for titrating laser settings [16, 17, 33]. Our study revealed that laser thermal damage caused by continuous-wave and micropulse laser modes differed (Figures 2(e) and 2(f)). Our results also suggested that laser power and exposure time titrations for the treatment of retinal disease should be performed using subthreshold micropulse photocoagulation and not continuous-wave laser photocoagulation.

Sublethal micropulse photothermal stimulation sites had cellular groups with sparse intercellular spaces that did not have any dead cells, even 24 h after laser irradiation, as determined using cell viability assays (Figure 2(d)). We presumed that these intercellular spaces represented the loss of cell-to-cell adhesion in the RPE layer, which may contribute to a dysfunctional blood retinal barrier in vivo, directly increasing fluid and molecule movement between the retina and choroidal tissues. Restoring the blood retinal barrier with photothermal stimulation may lead to improvements in macular edema. Future research on proteins related to RPE cell adhesion and RPE tight junctions (e.g., occludin, claudin, and zonula occludens-1) is needed in RPE cell layers.

Several studies have shown that heated tissue remaining viable after conventional continuous-wave laser treatment undergoes a stress response, which includes production of beneficial intracellular biological factors (e.g., PEDF, TSP-1, SDF1, and Hsp) with antiangiogenic and restorative functions [25, 30, 34]. Because Hsp may block the activity of apoptotic and inflammatory pathways that cause cellular damage [20–24], upregulation of Hsp70 expression induced by laser irradiation is thought to be important for reducing macular edema following clinical laser therapy [25, 26]. In the current study, sublethal micropulse photothermal stimulation was shown to induce upregulation of Hsp70, the major Hsp in mammalian cells. Moreover, expression of* Hsp70* mRNA, quantified by real-time qRT-PCR, was temporarily upregulated in preconditioned cultured cell layers for 12 h following laser irradiation. This change was not observed in the untreated control group. Immunostaining indicated that expression of Hsp70 proteins in cultured cell layers occurred concentrically around laser-irradiated sites. Additionally, Hsp70 protein production persisted for 24 h after sublethal laser irradiation. This study is the first to report Hsp70 expression and upregulation in cultured cells following sublethal micropulse laser energy application.

The beneficial effects of subthreshold micropulse photocoagulation for diabetic macular edema are not observed immediately in the clinical setting but begin to become evident 3 months following therapy [10, 13–17]. Therefore, sublethal micropulse photothermal stimulation that induces Hsp70 upregulation may facilitate RPE remodeling and contribute to improvements in macular edema.

Our study had several limitations. First, we examined a monolayer of cultured cells, which may not have the same vertical heat conduction properties as an intact eye. Secondly, ARPE-19 cells exhibit some characteristics and features that are different from those of human RPE cells. Therefore, the laser irradiation system developed and used in this study should be further examined in ARPE-19 cells and in human RPE cells derived from retinal tissue and induced pluripotent stem cells.

## 5. Conclusions

In conclusion, while the therapeutic mechanism underlying the efficacy of micropulse laser therapy has not yet been determined, our data showed that Hsp70 may be a trigger for improving RPE health and function. Moreover, Hsp70 may be used as a biological marker to determine therapeutic laser levels for subthreshold micropulse photocoagulation. In addition, testing laser irradiation systems in a cultured layer of human RPE cells is advantageous because changes in cell morphology, molecular composition, cellular transcriptomes, and cellular proteomes after sublethal photothermal stimulation can be precisely assessed using live imaging microscopy, DNA microarrays, and mass spectrometry techniques.

## Supplementary Material

Spots were made using the continuous-wave mode with a power of 250 mW, duration of 500 ms, and spot size of 200 μm. ARPE-19 cells are gradually changing ring-shaped form in periphery area of irradiate sites 2 hours after the ablation.
